# Numerical Investigation on the Performance of Compressible Fluid Systems in Mitigating Close-Field Blast Effects on a Fiber Circle

**DOI:** 10.3390/ma18102204

**Published:** 2025-05-10

**Authors:** Wei Zhu, Wenjin Yao, Jian Liu, Yu Zheng, Wenbin Li, Xiaoming Wang

**Affiliations:** 1 ZNDY Laboratory, Nanjing University of Science and Technology, Nanjing 210094, China; 12021059@njust.edu.cn (W.Z.); zhengyu@njust.edu.cn (Y.Z.); lwb2000cn@njust.edu.cn (W.L.); 202xm@163.com (X.W.); 2 Beijing Institute of Electronic System Engineering, Beijing 100854, China; 18810934039@163.com

**Keywords:** nanoporous material liquid system, energy absorption system, blast mitigation, numerical simulation, fiber-reinforced composite

## Abstract

Nanoporous material liquid systems (NMLSs) demonstrate promising potential for blast protection due to their high energy absorption density. This investigation numerically evaluated the use of NMLSs in mitigating blast effects on fiber–composite circular structures. The coupled Eulerian–Lagrangian method was employed to establish the numerical models of fiber alone, water–fiber, and NMLS–fiber, subjected to the internal close-field blast loading. The simulations focused on a widely studied NMLS, nanoporous silica particles immersed in distilled water. Four NMLSs, featuring varying particle-to-water ratios yet identical densities to that of water, were designed to modulate the energy absorption capacity while maintaining identical mass. These NMLSs were modeled by Equation of State (EOS) compaction. The dynamic responses of the fiber circles in the simulations were compared to evaluate the blast mitigation of different liquids. When the explosive mass was relatively small or medium, both the water and NMLSs exhibited blast mitigation. The NMLSs outperformed water because the energy absorption capacity caused a greater attenuation of blast pressure in the NMLSs. In the small-mass explosive cases, all four NMLSs could rapidly reduce the blast pressure to the infiltration pressure but their wave impedances decreased as the particle-to-water ratio increased, resulting in that a NMLS with greater energy absorption capacity, however, had inferior blast mitigation performance. When the explosive mass was relatively large, all the fiber circles experienced significant fiber failure and only the NMLS with the greatest energy absorption capacity exhibited blast mitigation.

## 1. Introduction

Nanoporous material liquid systems (NMLSs), consisting of nanoporous materials immersed in non-wetting liquids, are recently discovered energy absorption systems (EASs) [1,2,3]. At atmospheric pressure, the non-wetting liquid remains outside the nanopores due to the capillary effect. Once the external load pressure exceeds the threshold value, i.e., the infiltration pressure, the liquid molecules can rapidly infiltrate into the nanopores. The pressure then rises gradually in a plateau-like manner until all the nanopores are filled. Conversely, it is difficult for the liquid to exit the nanopores as the pressure is unloaded. Due to the inelastic volumetric deformation caused by this infiltration process, NMLSs are compressible and able to efficiently absorb energy primarily by converting external work into solid–liquid interfacial energy [4,5,6]. Owing to their ultrahigh specific surface areas (100–1000 m^2^/g) [7], nanoporous materials exhibit exceptional energy absorption efficiency, typically ranging from 10 to 100 J/g [8,9,10]. This is significantly higher than that of traditional EASs, such as metallic and polymeric foams, which generally perform at levels below 10 J/g [11]. Therefore, NMLSs holds great promise for use in many protective structures designed to mitigate loads from collision, impact, explosion, and other events.

Extensive studies have been conducted to investigate the energy absorption characteristics of NMLSs [8,9,12,13,14]. Kong et al. [12] performed quasi-static compression experiments on a NMLS consisting of nanoporous silica particles immersed in deionized water, which was sealed in a compressible container, to determine the thermal effects on the energy absorption efficiency. It was found that as the temperature increased, although the infiltration pressure decreased only slightly, the system recoverability could be significantly improved. Surani et al. [13] investigated the performance of this NMLS under dynamic loadings through a Hopkinson bar experiment. The energy absorption efficiency was determined to be 41 J/g, which is significantly higher than the quasi-static value, likely due to the effect of internal friction. Kong et al. [8] further experimentally studied the effect of chemical admixtures, particularly sodium chloride, on the energy absorption behavior. The experimental results demonstrated that the addition of NaCl led to higher infiltration and outflow pressures, and increasing NaCl concentration was beneficial to the energy absorption efficiency. Sun et al. [14] conducted low-speed impact experiments on a different NMLS, specifically the zeolite β/water system, which exhibited a quasi-static energy absorption efficiency of 3.24 J/cm^3^. Using a drop hammer setup, they discovered that the NMLS demonstrated a substantial cushioning effect, effectively reducing the maximum peak force while extending the reaction time.

Given the high energy absorption efficiency of NMLSs, many researchers [10,15,16,17,18,19,20] explored their application value in enhancing the traditional EASs. Chen et al. [10] performed quasi-static compression experiments on a steel tube filled with a NMLS. The energy absorption efficiency, both on a mass and volumetric basis, was significantly improved because the work performed by the axial load was dissipated not only through conventional cell-wall buckling but also via extended yielding and pressure-induced infiltration. Li et al. [16] compared the performance of the steel tubes either filled with a NMLS or a solid foam through quasi-static compression and dynamic impact tests. Results showed that the strengthening coefficient for the NMLS was 3.8, much higher than that of the best foam. This enhancement was attributed to the liquidity of the NMLS, which provided “perfect bonding” with the tube wall. Sun et al. [18] conducted a similar study, with additional consideration given to the influence of tube properties on the energy absorption mechanism of metallic tubes filled with a NMLS. Liu et al. [19,20] experimentally and numerically studied the quasi-static crush behavior of a hollow microtruss filled with a NMLS. It was found that filling NMLS into a relatively thin microtruss improved the load capacity as well as the energy absorption efficiency more than two times. This enhancement came from not only the volumetric plasticity of the NMLS but also the plastic deformation enhancement of the microtruss. In their numerical study, Equation of State (EOS) compaction, which was originally designed for modeling the compaction of ductile porous materials, was employed to phenomenologically describe the infiltration process of NMLS. This method was also employed by Chen et al. [10] and Sun et al. [18], and its reliability was confirmed by the corresponding experimental results.

Although many researchers [5,9,21,22,23] considered NMLSs suitable for mitigating short-duration impulse energy, such as blast loading, the blast mitigation performance of NMLSs has been rarely studied. Among the limited studies, Xu et al. [22] carried out molecular dynamics simulations to investigate the effects of several key parameters (e.g., impact velocity, nanopore size, and pore composition) on the mitigation performance of a NMLS on impact/blast energy, which were qualitatively validated by a parallel blast experiment on a zeolite/water system. It should be noted that this study focused on the molecular-level response of the NMLS itself and did not address the macroscopic responses of protective structures and the protected structures, which are critical for blast-mitigation considerations.

In the current investigation, numerical simulations were carried out to evaluate the use of a NMLS in mitigating the blast effects on a fiber–composite circle. The coupled Eulerian–Lagrangian (CEL) method was employed to establish numerical models for three types of structures, the fiber circle alone, water–fiber circle, and NMLS–fiber circle, subjected to the internal, close-field blast loading from the detonation of three different masses of TNT charges. The NMLS was modeled using EOS compaction to phenomenologically describe its microscale energy absorption behavior. Comparisons were made between the dynamic responses of the fiber circles in these structures to quantitatively evaluate the benefits derived from the energy absorption capacity of the NMLS. Pressure attenuation characteristics in the different liquids were analyzed to find the reason for their difference in blast mitigation performance.

## 2. Numerical Methodologies

### 2.1. Introduction of Numerical Models

Fiber–composite circles are extensively utilized for the emergency containment and disposal of improvised explosive devices (IEDs) discovered in public areas. These structures help protect individuals and equipment from explosion hazards, such as high-speed fragments and blast waves, originating from the IEDs. This investigation considered a scenario where a cylindrical TNT explosive detonated within a fiber–composite circle. The blast waves produced by the explosion would compress the circle along the radius direction and stretch the fibers along the circumferential direction, resulting in global deformation and hoop tensile stresses. These blast effects were critically dependent on the intensity of the blast loading exerted on the fiber circle, and sufficiently intense blast loading could cause fiber failure. Placing a hollow cylindrical liquid layer inside the fiber circle was anticipated to mitigate the blast effects on the fiber circle. The blast mitigation performance of this liquid layer could be evaluated by determining the response of the fiber circle.

The current simulations considered three different liquid–fiber composite structures, named AP, WP, and NP. Structure AP contained only the fiber circle without any liquid and served as the baseline. Structure WP used an incompressible fluid, water. Water cannot significantly absorb blast energy, but it can contribute to sharing a portion of the blast-induced momentum by increasing the effective mass of the target. Comparing WP with AP would help us understand whether the momentum extraction effects contribute to blast mitigation. Structure NP replaced the water with a NMLS of the same density, thereby introducing the ability of energy absorption into the liquid while maintaining an identical mass. Comparing NP with WP would help us understand how the energy absorption of a liquid affects its blast mitigation performance.

**Figure 1:**
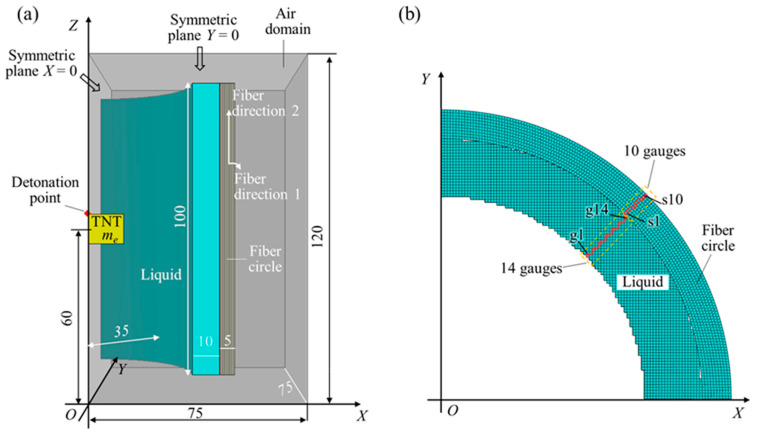
(**a**) Composition and geometric dimensions of the numerical model. (**b**) A 14-gauge set in the liquid and 10-gauge set in the fiber circle; these gauges were positioned at the mid-height of the liquid–fiber structure.

The explosion of a charge in the liquid–fiber structure involves a series of processes, including the detonation of explosives, shock wave propagation in the air and the liquid, and fluid–structure interaction. To characterize these processes, the CEL method was employed to establish the finite-element models in the Abaqus/Explicit, as shown in Figure 1. The Eulerian algorithm was used to represent the air domain, TNT explosive, NMLS, and water, while the Lagrangian algorithm was used to represent the fiber–composite circle. Considering the symmetry of the current research scenario, quarter models were utilized to improve computational efficiency. Symmetric boundary conditions were applied on the planes X = 0 and Y = 0, while flow-out boundary conditions were applied on the other external surfaces of the Eulerian domain. This Eulerian domain had dimensions of 75 mm × 75 mm × 120 mm. Both the Eulerian domain and the Lagrangian bodies were meshed with eight-node reduced integration solid elements, specifically EC3D8R for the Eulerian domain and C3D8R for the Lagrangian bodies. To suppress hourglass modes, the integral viscoelastic form of hourglass control was applied to C3D8R elements, whereas the pure viscous form of hourglass control was applied to EC3D8R elements [24]. A general contact setting was applied to the entire numerical model. During the analysis, Abaqus automatically identified the contact pairs between different materials and within the same material, including the Eulerian–Lagrangian contact interface, and handled the interactions between these contact pairs.

A fiber circle is typically prepared by continuously rolling a fiber pre-preg. Most commercial ultra-high-molecular-weight polyethylene (UHMWPE) fiber pre-pregs consist of two or four plies of unidirectional fiber films stacked orthogonally, resulting in a thickness of approximately 0.1 mm. In the numerical models, the rolling structure was not taken into account. Instead, the fiber circle was modeled as a combination of several layers of individual fiber pre-pregs with a stacking sequence of [0°/90°/0°/90°…], and each layer was circumferentially closed. The two fiber directions, 0° for direction 1 and 90° for direction 2, as shown in Figure 1a, were aligned with the circumferential and height directions of the fiber circle, respectively. The fiber–composite circle exhibited orthotropic behavior with respect to these two fiber directions. Consequently, the fiber circle was symmetric with respect to the planes X = 0 and Y = 0, thereby supporting the use of quarter models. Additionally, a larger thickness of 0.5 mm (versus 0.1 mm) was used for a single fiber pre-preg to reduce the total number of elements and increase the stable time increment, thereby ensuring the computational feasibility and efficiency. This pre-preg can be considered a merger of several layers of commercial pre-pregs. The fiber circle had an inner radius of 45 mm and a height of 100 mm.

A liquid is generally contained within a container to form a fixed shape. A low-density, thin-walled polymer container is suitable for holding the water and NMLS in the current composite structure. However, the numerical model did not include this container because it has a minimal impact on the explosion effects, and its exclusion simplifies the model. Therefore, the liquid layer directly contacted the inner side of the fiber circle and had the same height as the fiber circle. This liquid had an inner radius of 35 mm, corresponding to a thickness of 10 mm. A cylindrical TNT charge with a fixed radius of 12.5 mm was used to produce the blast loading. This charge was positioned coaxially with the fiber ring, and their geometric centers coincided. A detonation point was set at the top of the charge.

In the simulations, the geometries of the liquid–fiber structures were kept fixed (Figure 1a), while the material types of the liquid layer and the mass of the TNT charge were varied (Table 1). The simulations considered three different masses (i.e., different heights) of TNT charges, which were distinguished by the first letter of the configuration name. Letters S, M, and L correspond to small (3.80 g), medium (7.68 g), and large masses (11.52 g), respectively. Varying the mass of the charge aimed to change the response level of the fiber circle by inducing different intensities of blast loadings, so that comprehensive comparisons could be made between AP, WP, and NP. Since the energy absorption capacity of the NMLS is critically dependent on the total pore volume of the nanoporous particles, increasing the mass ratios *r_p_* of the nanoporous particles to water improved the energy absorption capacity of the NMLS. The simulations considered four different NMLSs, named N1, N2, N3, and N4, which had *r_p_* values of 1:1, 1:2, 1:3, and 1:4, respectively.

### 2.2. Material Models

The current numerical models consist of five different materials, i.e., NMLS, water, fiber composite, air, and TNT. Different material models were employed to describe their dynamic behaviors.

#### 2.2.1. Nanoporous Material Liquid System

The current investigation considered a widely studied NMLS, Fluka 100 C8 nanoporous silica particles (Sigma-Aldrich, St. Louis, MO, USA), immersed in distilled water. The Fluka 100-C8 particle size ranged from 15 to 35 µm. The average pore size was 7.8 nm, and the specific pore volume was 0.55 cm^3^/g [8]. Since the density of non-porous silica is 2.2 g/cm^3^, it can be calculated that Fluka 100-C8 particles have a density of ~1 g/cm^3^, which is roughly identical to that of water. As a result, the density of this NMLS should be approximately 1 g/cm^3^ regardless of the value of *r_p_*, considering that water cannot infiltrate into the nanopores nor dissolve the particles at atmospheric pressure. This property provided great benefits for making fair comparisons between water and NMLSs of varying *r_p_* since the masses of these different liquids could remain almost the same, which is crucial for evaluating the blast mitigation performance.

**Figure 2:**
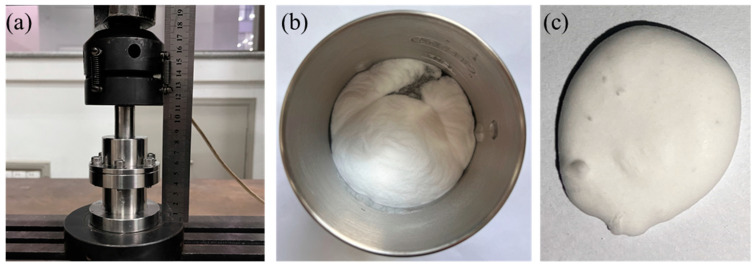
(**a**) Compressive tests on NMLS sealed in a steel chamber, (**b**) preparation of the NMLS using a high-speed mixer, and (**c**) the NMLS after mixing.

To determine the influence of *r_p_* on the compressive response of this type of NMLS, quasi-static mechanical testing was carried out. Following the approach used by Kong et al. [12] and Sun et al. [14], the NMLS sample was sealed in a steel chamber with an inner diameter of 20 mm and compressed through a sliding steel piston at a constant rate of 3.6 mm/min (Figure 2a). To obtain a more uniformly mixed NMLS, the particles and water were put into a high-speed mixer and stirred before being sealed in the chamber (Figure 2b,c). The NMLSs of three different *r_p_* values, 1:2, 1:3, and 1:4, were prepared and tested. All the samples had the same mass of 8 g.

**Figure 3:**
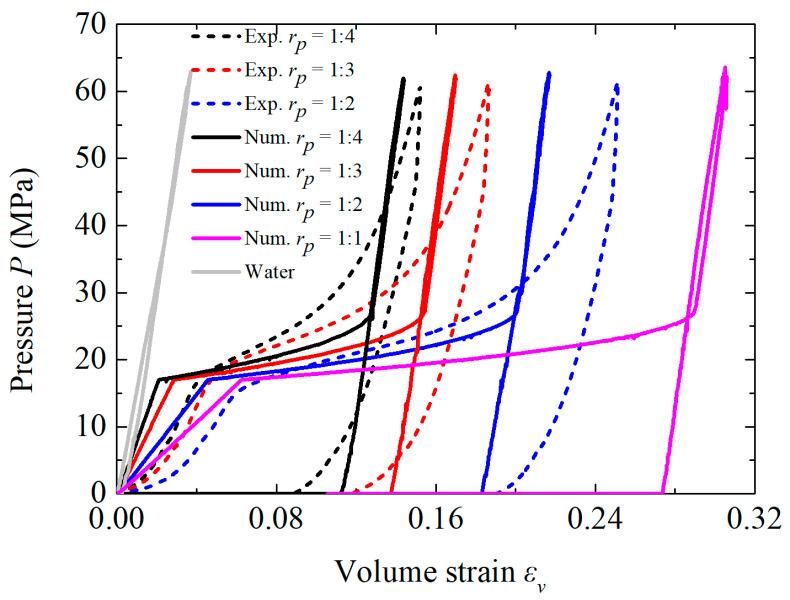
The *P*–*ε_v_* curves for NMLSs of different *r_p_* values, including data from the experiments and the numerical simulations.

The obtained curves of pressure *P*–volumetric strain *ε_v_* are shown in Figure 3. Consistent with the previous results in the literature [6,9,25,26,27], the compressive behavior of the NMLS generally exhibited three stages: the elastic deformation, the infiltration of the liquid molecules into the nanopore, and the densification. The infiltration process, corresponding to the plateau segment of the curves, began when the applied pressure exceeded the capillary pressure of the nanopore *P*_in_ and concluded once all of the nanopores were filled with the liquid. The pressures *P*_in_ for *r_p_* = 1:2, 1:3, and 1:4 were approximately 17.4, 18.1, and 15.7 MPa, respectively, which are close to the one (17 MPa) reported in the previous studies [13,28]. The change in *ε_v_* corresponding to the infiltration process increased with *r_p_*, leading to an improved energy absorption capacity. Additionally, *r_p_* barely affected the bulk modulus of the NMLS after densification, as indicated by the slope of the unloading part of the curves. However, the bulk modulus at the initial stage of the elastic deformation decreased with increasing *r_p_*.

The FE model cannot explicitly characterize the infiltration process, which occurs at the microscale. However, this process can be phenomenologically described by EOS compaction, which is originally designed for modeling the compaction of ductile macro-porous materials [29,30]. Chen et al. [10] and Liu et al. [20] have proved that this equivalent approach is sound in their investigation on the compressive response of steel tubes enhanced by NMLSs. This EOS connects the pressure *p* of the porous material with the pressure *p_s_* of the solid matrix via a scalar variable *α* and is expressed as

(1) $$p(\alpha ,\rho ,E_{m})=\frac{1}{\alpha }p_{s}(\rho_{s},E_{m}),$$

where *ρ* and *ρ_s_* are the current density of the porous material and the solid matrix, respectively, while *E_m_* is the specific internal energy of the porous material as well as the solid matrix because the surface energy of the pores is neglected [24]. *α* is defined as the ratio of *ρ_s_* to *ρ*. This EOS had a plastic branch and multiple elastic branches, corresponding to elastic unloading from partially compacted states. In the current simulations, the initial value of *E_m_* was zero for NMLSs. Their solid matrix, which corresponds to the state where all nanopores are completely filled with water, was modeled using the Mie–Grüneisen EOS in a linear *Us*–*Up* form [20,24].

(2) $$p_{s}=\frac{\rho_{0}{c_{0}}^{2}\eta }{{\left(1-s\eta \right)}^{2}}(1-\frac{\Gamma_{0}\eta }{2})+\Gamma_{0}\rho_{0}E_{m},$$

where *ρ*_0_ and *c*_0_ are the reference density and sound speed, respectively, *s* is the slope of the fitting curve, Γ_0_ is the Grüneisen coefficient, and *η* is defined as *η* = 1–*ρ*_0_/*ρ_s_*. The material parameters for the NMLSs are shown in Table 2. The reference sound speed *c*_0_ was identical to that of water, while *s* and Γ_0_ were simplified to zero, as per Liu et al. [20]. The reference sound speed in the porous material *c_p_*_0_ increased as *r_p_* decreased to stay consistent with the experimental observations in Figure 3. The current material model did not take the strain-rate effects into consideration, and thus, the infiltration pressure *P_in_* used the quasi-static value of 17 MPa.

**Figure 4:**
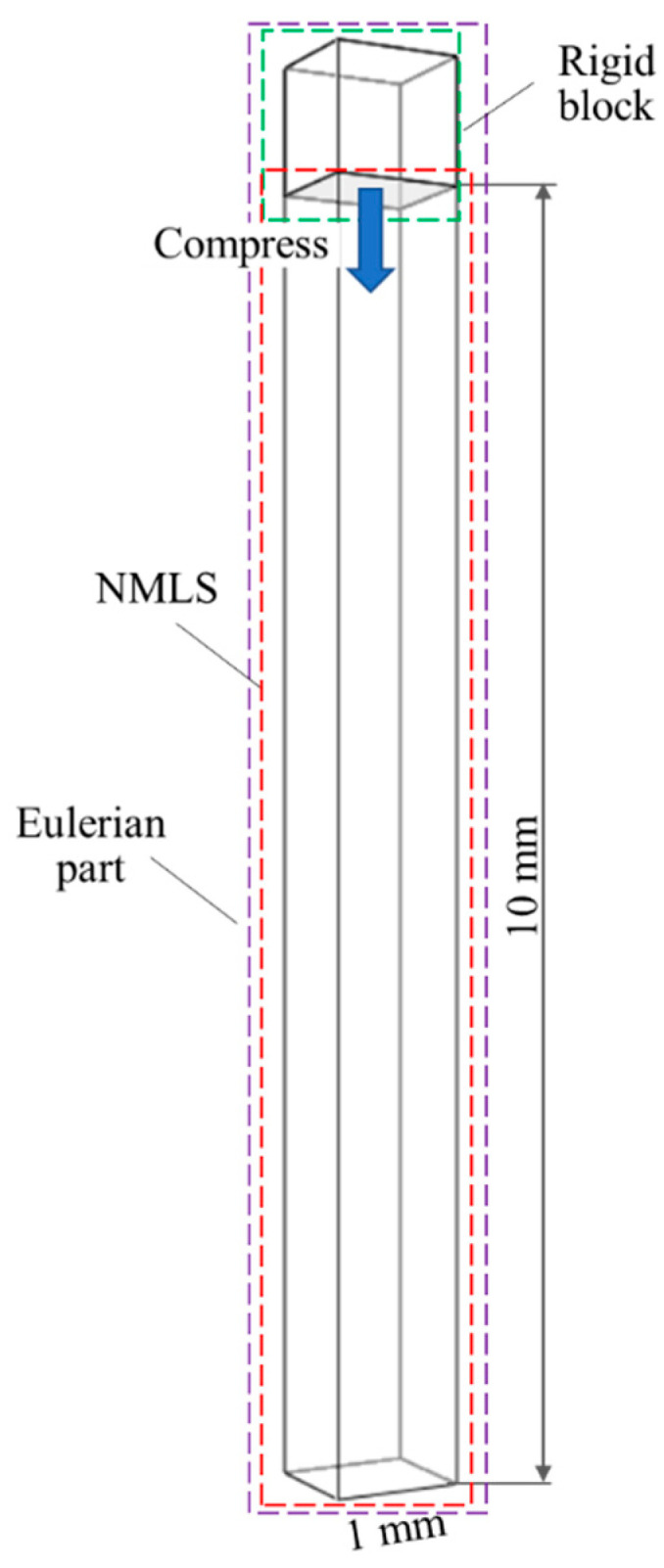
The numerical model of uniaxially compressing the NMLS.

Numerical simulations of the NMLS under uniaxial compression were conducted to validate this phenomenological method. As shown in Figure 4, the model comprised a 1 mm × 1 mm × 11 mm rectangular Eulerian domain and a rigid block. The Eulerian domain was discretized using 8-node solid elements of 1 mm in size, and the NMLS had a length of 10 mm. The rigid block initially compressed this NMLS until a pressure of ~62 MPa and then retracted. The normal degrees of freedom on each external surface of the Eulerian domain were fixed to simulate the confinement effect of a steel chamber on the NMLS. The obtained *P*–*ε_v_* curves for the NMLSs of different *r_p_* and water are shown in Figure 3. The material model did not account for the strong non-linear behaviors of the NMLS, resulting in some deviations of the numerical curves from the corresponding experimental ones. Nevertheless, these curves from the simulations and experiments exhibited quite similar compressive responses in terms of the three stages, indicating that the material model for the NMLS was reliable in representing its macro-mechanical behaviors and could be used for the subsequent numerical simulations of blast mitigation.

#### 2.2.2. Fiber-Reinforced Cross-Ply Composite

The simulations assumed that the fiber circle used the commercial UHMWPE pre-preg Dyneema^®^ HB26 (DSM, Urmond, the Netherlands), whose mechanical properties have been well determined by the experiments in the literature [31,32]. HB26 exhibits a strain-rate-dependent anisotropic elastoplastic constitutive relationship. In the simulations, this relationship was mainly described by the classic elastic constitutive relationship for fiber composites and the yield surface *F* based on a quadratic yield function proposed by Chen et al. [33]. This yield surface is defined by

(3) $$F=\sqrt{a_{11}{\sigma_{11}}^{2}+a_{22}{\sigma_{22}}^{2}+a_{33}{\sigma_{33}}^{2}+a_{44}{\sigma_{12}}^{2}+a_{55}{\sigma_{23}}^{2}+a_{66}{\sigma_{13}}^{2}}-\bar{\sigma }({\bar{\varepsilon }}^{p},{\dot{\bar{\varepsilon }}}^{p}),$$

where $\sigma_{ij}$ is the stress component, $a_{ij}$ denotes the plasticity coefficients, and $\bar{\sigma }({\bar{\varepsilon }}^{p},{\dot{\bar{\varepsilon }}}^{p})$ is the strain-rate-dependent yield strength. The Young’s modulus and shear modulus are also strain-rate dependent. One of our previous studies [34] provided a detailed introduction to this constitutive model and the corresponding material parameters for HB26, which are not repeated in this paper for simplicity.

The current material model only considered one key failure mode, i.e., fiber tensile failure. The fibers would break brittlely once one of the tensile stresses, $\sigma_{11}$ and $\sigma_{22}$, along the fiber directions exceeded the corresponding fiber tensile strength, $X_{ft1}$ or $X_{ft2}$.

(4) $$\sigma_{11}\geq X_{ft1},\;\mathrm{or}\;\sigma_{22}\geq X_{ft2}.$$

Russell et al. [31] carried out mechanical experiments on the fibers of HB26 laminates across a wide range of strain rates, from 10^−4^ to 10^3^ s^−1^. The experimental results indicate that the fiber tensile strength increases with the strain rate at low strain rates (below 10^−1^ s^−1^) and remains nearly constant at higher strain rates. Therefore, in the simulations, the fiber tensile strength was a function of the strain rate. $X_{ft1}$ is defined as follows, for example,

(5) $$\begin{array}{l} X_{ft1}=X_{ft,0}(1+0.075\log_{10}\frac{{\dot{\varepsilon }}_{11}}{{\dot{\varepsilon }}_{11,0}}),\;\mathrm{when}\;{\dot{\varepsilon }}_{11}<0.1{\;s}^{-1},\;\mathrm{and}\\ X_{ft1}=1050\;\mathrm{MPa},\;\mathrm{when}\;{\dot{\varepsilon }}_{11}\geq 0.1{\;s}^{-1} \end{array}$$

where $X_{ft,0}$ is the reference fiber tensile strength at the reference strain rate ${\dot{\varepsilon }}_{11,0}$. In the simulations, $X_{ft,0}$ and ${\dot{\varepsilon }}_{11,0}$ were 913 MPa and 0.001 s^−1^, respectively. Once the fiber failure criterion in Equation (4) was met, the corresponding element was deleted.

#### 2.2.3. Air and TNT Explosive

The numerical models employed an EOS ideal gas with parameters obtained from a previous publication [35] (Table 3) to describe the air. The EOS Jones–Wilkins–Lee (JWL) with parameters obtained from a previous publication [34] (Table 4) was applied for the TNT explosive.

### 2.3. Blast Loading and Its Mesh Dependence

**Figure 5:**
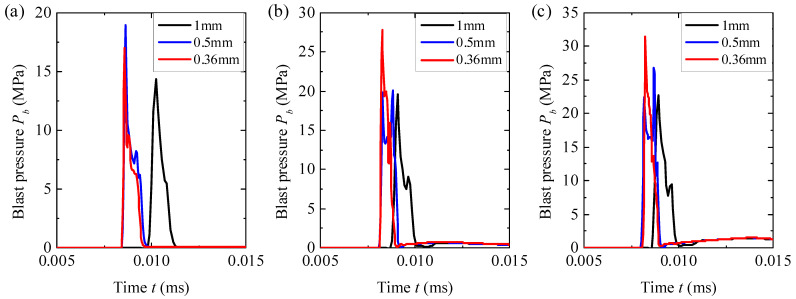
Histories of free-field blast pressure *P_b_* at the stand-off distance of 45 mm; these histories were extracted from the simulations using different mesh sizes for the cases of (**a**) *m_e_* = 3.80 g, (**b**) *m_e_* = 7.68 g, and (**c**) *m_e_* = 11.52 g.

A mesh-dependence study was performed on the Eulerian domain to determine an appropriate element size. Three different sizes (1, 0.5, and 0.36 mm) were chosen for the side length of the Eulerian elements. The liquid–fiber structure was removed from the simulations so that the free-field blast-wave pressures could be obtained to determine the influence of the element size on the blast loading. The pressure (*P_b_*) histories at the stand-off distance of 45 mm (i.e., the location of the fiber circle) extracted from those simulations are compared in Figure 5.

Using the 1 mm mesh size led to significant deviations in the pressure histories from those using 0.5 and 0.36 mm for all three cases of different TNT masses *m_e_*. Reducing the element size from 0.5 mm to 0.36 mm still made some differences in the peak pressure but barely changed the arrival time and impulse duration of the blast waves. These differences in peak pressure were 10.2%, 28.1%, and 15.0% for *m_e_* = 3.80, 7.68, and 11.52 g, respectively. However, the Eulerian part using a 0.5 mm mesh size consisted of 5,400,000 elements, and the fiber circle added another 299,000 elements (with the same size of 0.5 mm) into the numerical model. This led to a total element count of 5,699,000, which already corresponded to a significant computational demand. Using a mesh size smaller than 0.5 mm (such as 0.36 mm) would not only substantially increase the total number of elements but also significantly reduce the stable time increment, causing difficulties in completing the computation. Therefore, the numerical models adopted a global mesh size of 0.5 mm. Given that the main goal of this investigation was to ensure fair comparisons between different liquid–fiber structures, the selected mesh size was generally suitable.

## 3. Numerical Results and Analyses

For a particular charge mass *m_e_*, comparisons were made between the structures with different liquids in terms of fiber failure, hoop tensile stress, and energy obtained by the fiber circle. The pressure attenuation characteristics in the liquids were then compared to identify the reasons for their different blast mitigation performances.

### 3.1. Using the TNT Charge of 3.80 g

**Figure 6:**
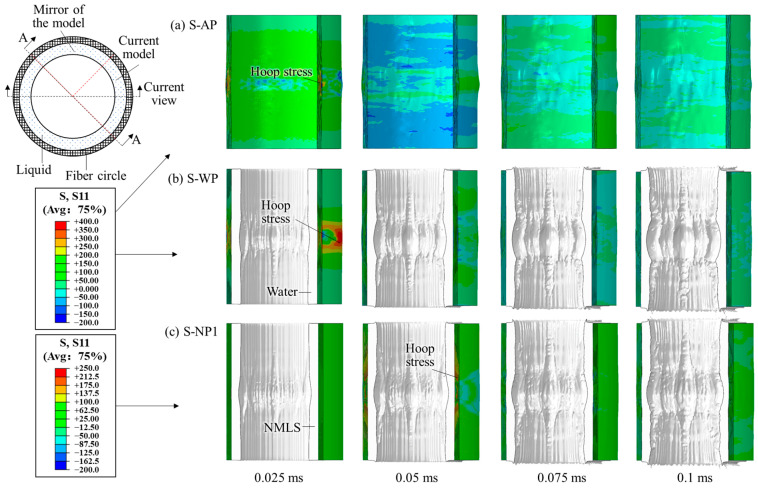
Dynamic response of the structures (**a**) AP, (**b**) WP, and (**c**) NP1 subjected to the blast loading produced by the 3.8 g TNT charge. A mirror of the current numerical model is also shown.

Subjected to the blast loading from a 3.8 g TNT charge, no fiber failed in all six structures. The dynamic responses of the AP, WP, and NP1 are shown in Figure 6. Because the fiber circle was closed in the circumferential direction, the blast loading developed fiber tensile stress mainly along the hoop direction, further causing small radial bulges. For the structures WP and NP, the blast loading was initially exerted on the liquids, leading to the motion and compression of the liquids. The blast loading was then transmitted through the liquid–fiber interface into the fiber circle.

**Figure 7:**
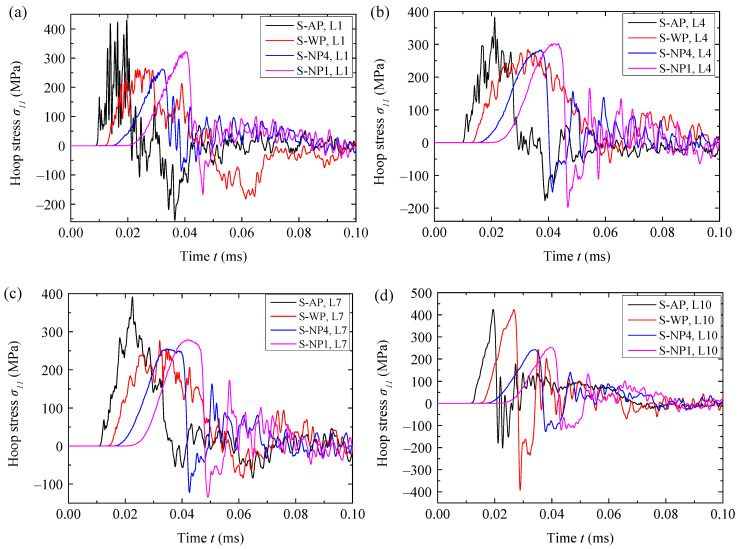
Histories of the hoop stress *σ*_11_ at the gauges (**a**) L1, (**b**) L4, (**c**) L7, and (**d**) L10 of the different structures.

The histories of the hoop stress *σ*_11_ in the different fiber layers of the structures were compared and shown in Figure 7. The hoop tensile stress in the fiber circle of structure AP rose to a peak in a highly oscillatory manner, especially in these fiber layers close to the blast-facing surface, because the blast loading was easily unloaded by rarefaction waves transmitted from this surface. In the rising segment for structure WP, the tensile stress also oscillated but with a much smaller amplitude. This is because the rarefaction waves could quickly propagate into the fiber circle through the water layer, given that water has a relatively large wave speed of 1500 m/s. By contrast, the tensile stress in the fiber circle of structure NP rose in a smoother manner, indicating that the unloading effects were weakened by the inelastic volumetric deformation corresponding to the infiltration process. Both the water and the NMLS delayed the arrival time of blast loading into the fiber circle. The NMLS caused a longer delay compared to water, and the delay time increased slightly with an increase in the mass ratio *r_p_* of nanoporous particles to water.

**Figure 8:**
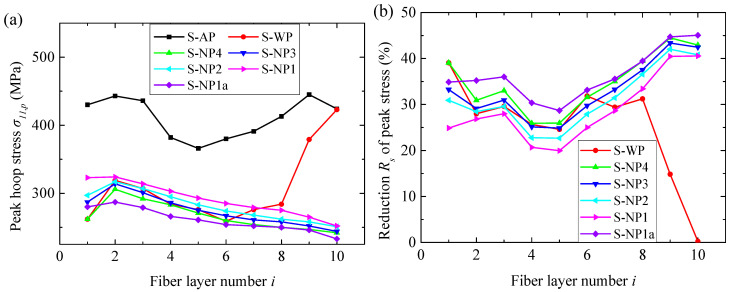
(**a**) The peak hoop stresses *σ*_11,*p*_ at the gauges of each fiber layer in cases of 3.80 g of TNT and (**b**) the reduction *R_s_* in peak stress caused by the water and four NMLSs.

The peak hoop stresses *σ*_11,*p*_ at the gauges of each fiber layer are shown in Figure 8a. In term of *σ*_11,*p*_, both the water and NMLSs mitigated the blast effects on the fiber circle compared to structure AP. To quantitatively characterize the mitigation performance, the reduction *R_s_* in *σ*_11,*p*_ was defined and calculated for each fiber layer.

(6) $$R_{s}=\frac{\sigma_{11,p}^{A}-\sigma_{11,p}^{L}}{\sigma_{11,p}^{A}}\times 100\%,$$

where $\sigma_{11,p}^{A}$ and $\sigma_{11,p}^{L}$ are the peak hoop stress of the structures without liquid (i.e., AP) and with a liquid (i.e., WP and NP), respectively. *R_s_* caused by the water and NMLSs are shown in Figure 8b. The water layer caused a maximum reduction *R_s_* of 39.1% occurring in the first fiber layer. This reduction remained at high values for the first eight fiber layers but dramatically decreased in the last two layers. The four NMLSs caused reductions *R_s_* ranging from 19.9% to 45.0%. In terms of *R_s_*, the NMLSs had blast mitigation performance comparable to that of water in the first six fiber layers, yet significantly outperformed water in the last three layers. When considering the influence of the mass ratio *r_p_* of NMLSs, the results did not follow the expected trend of *R_s_* increasing with *r_p_*. On the contrary, *R*_s_ basically increased in the order of NP1, NP2, NP3, and NP4, i.e., the NMLS with a smaller energy absorption capacity, however, had better migration performance.

**Figure 9:**
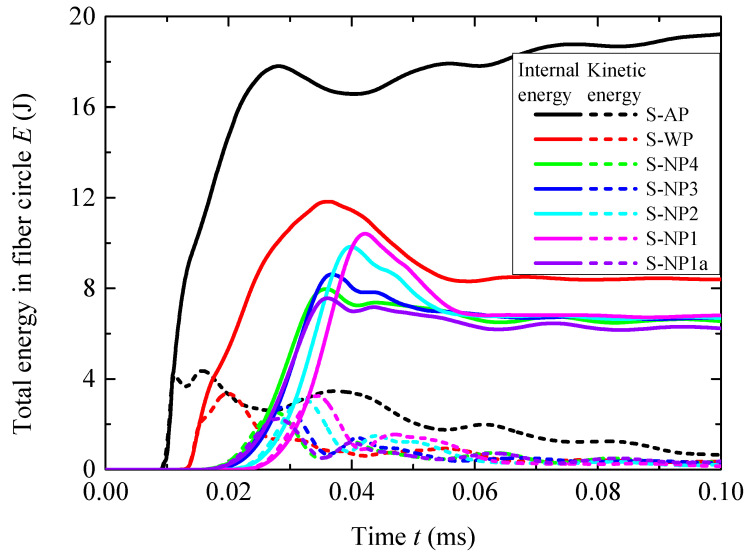
Histories of the internal energy *E_i_* and kinetic energy *E_k_* in the fiber circles for cases of 3.80 g of TNT.

To estimate the overall blast mitigation performance of different liquids, the histories of the total internal energy *E_i_* and kinetic energy *E_k_* obtained by the fiber circle were extracted from the simulations. The history curves are shown in Figure 9, and the peaks *E_ip_* and *E_kp_* for each curve are shown in Table 5. *E_i_* for structure AP continued to grow intermittently after its initial peak because a minor portion of the blast pressure leaked into the interior of the fiber circle. Hence, *E_ip_* was taken at the initial peak. Both the water and the NMLSs reduced the energy obtained by the fiber circles. Reductions *R_i_* and *R_k_* were defined for *E_ip_* and *E_kp_*, respectively, using a similar method for peak hoop stress (Equation (6)). The water had an *R_i_* of 33.7% and an *R_k_* of 23.2%. All four NMLSs outperformed the water in overall blast mitigation performance; the values of *R_i_* and *R_k_* for these NMLSs ranged from 41.6% to 55.2% and from 25.5% to 41.7%, respectively. This result indicates that the energy absorption capacity of the NMLSs reduced the blast energy transferred into the fiber circle. Both *R_i_* and *R_k_* for the NMLSs decreased as *r_p_* increased, i.e., improving the energy absorption capacity of NMLSs, however, transferred more blast energy into the fiber circle. This influence of *r_p_* on the overall blast mitigation performance is consistent with that on the reduction in peak hoop stress.

**Figure 10:**
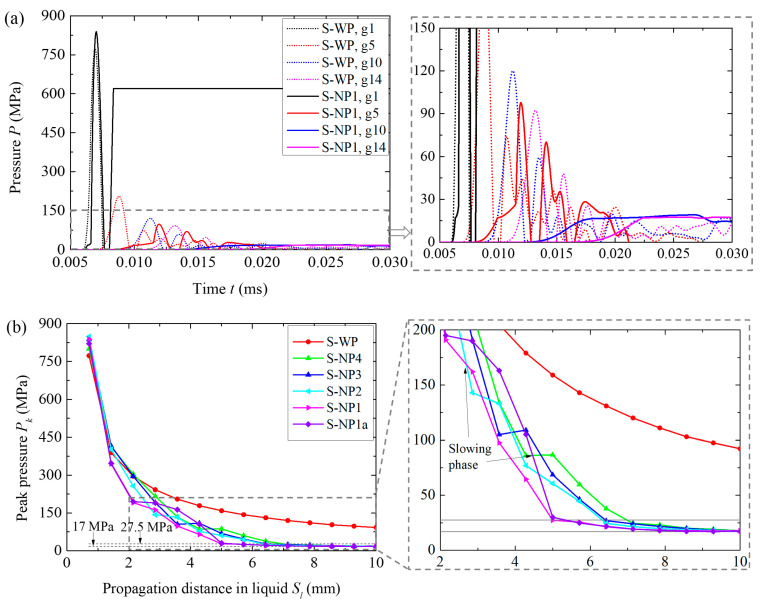
(**a**) Histories of the blast pressure *P* and (**b**) attenuation of the peak pressure *P_k_* in the different liquids in the cases using a 3.80 g TNT.

The attenuation characteristics of the blast pressure *P* in the different liquids were compared to verify whether the energy absorption capacity of the NMLS helped mitigate the blast loading in the liquid. The pressure histories at the different gauges (corresponding to different propagation distances *S_l_*) of water and NMLSs are shown in Figure 10a. In the water, *P* dramatically increased to the peak. By contrast, in the NMLS, a platform segment occurred once *P* exceeded 17 MPa, corresponding to the compaction of the NMLS. The arrival times of *P* to different *S_l_* were gradually delayed in the NMLS compared to those in the water, consistent with the results of the arrival time for the hoop stress of the fiber circles in Figure 7a.

The peak pressures *P_k_* varying with *S_l_* in the different liquids are shown in Figure 10b. In the water, *P_k_* decayed in an exponential form due to the constant wave speed, consistent with the previous studies [36,37]. By contrast, in the NMLS, *P_k_* experienced a slowing phase during its fast decay, e.g., at an *S_l_* of 2 to 3 mm for N1, and increasing *r_p_* led to an earlier onset of this slowing phase. This slowing phase was likely caused by the complex elastoplastic response of the NMLS, which induced multiple pressure waves of different propagation speeds. Nevertheless, in the NMLS, *P_k_* generally decayed faster than in the water, and the higher the value of *r_p_*, the faster the overall decay rate. When approaching the liquid–fiber interface, the *P_k_* in all the four NMLSs tended to the same value of 17 MPa (i.e., the infiltration pressure *P_in_*), which was only approximately 18.4% of *P_k_* (92.3 MPa) near the interface in the water. These results of pressure attenuation in the different liquids confirm that the energy absorption capability of the NMLS effectively contributed to mitigating blast loading. However, the NMLS of a large *r_p_*, such as N1, did not demonstrate a distinguished advantage in the pressure near the liquid–fiber interface compared to the NMLS of a small *r_p_*, such as N4, because the energy absorption capacity of N4 was already sufficient to reduce the blast pressure produced by the 3.68 g TNT to *P_in_*; i.e., the energy absorption potential of other NMLSs was not fully utilized.

Since the peak pressure *P_k_* near the liquid–fiber interface was almost identical for the four NMLSs, the blast mitigation performance of the NMLSs in the current cases using a 3.68 g TNT must be influenced primarily by another factor. It is evident that the blast pressure transmitted into the fiber circle depends not only on the pressure within the liquid but also on the wave impedance matching at the liquid–fiber interface. In the simulations, the UHMWPE fiber composite had a density $\rho_{f}$ of 0.98 g/cm^3^ and an out-of-plane Young’s modulus $E_{fo}$ of 3.62 GPa, resulting in a wave impedance $\sqrt{E_{fo}\rho_{f}}$ of 1884 t/(s·m^2^). In contrast, water had a wave impedance of 1500 t/(s·m^2^). The initial wave impedances of N1, N2, N3, and N4 were calculated to be 400, 500, 800, and 1000 t/(s·m^2^), respectively. Given that the *P_k_* values near the liquid–fiber interface for all NMLSs only slightly exceeded 17 MPa, the reflection and transmission of pressure waves at the interface should depend on these initial wave impedances. All the liquids had smaller wave impedances than the fiber composite, leading to enhanced pressure after transmission from the liquid into the fiber circle. The lower the liquid’s wave impedance, the greater this enhancement effect should become. The NMLS of a larger *r_p_* had a lower initial wave impedance; thus, it caused more significant enhancement at the liquid–fiber interface. This is likely the primary reason for its inferior blast mitigation performance.

To verify this reason, an additional simulation was conducted using an “artificial” NMLS, named N1a. N1a had all the same material properties as N1 except the initial sound speed *c*_0_, which was changed from 400 to 1000 m/s to remain the same as that of N4. The results of N1a are also included in Figure 8, Figure 9 and Figure 10 and Table 5. The *P_k_*–*S_l_* for N1a only deviated from that of N1 in the range of 2 mm < *S_l_* < 5 mm, i.e., at the slowing phase, while they were almost identical at other distances (Figure 10b), indicating that the pressure attenuation of NMLS mainly depended on the energy absorption capacity. Regarding the blast mitigation performance, N1a outperformed N4 in both the reduction in peak hoop stress and the energy transferred into the fiber circles, as expected. These results demonstrate that it was the difference in the wave impedance of the NMLSs that resulted in varying blast mitigation performances.

### 3.2. Using the TNT Charge of 7.68 g

**Figure 11:**
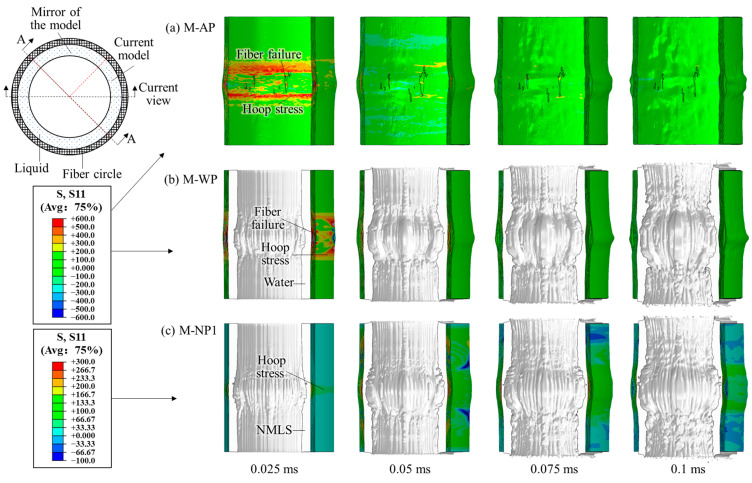
Dynamic response of the structures (**a**) AP, (**b**) WP, and (**c**) NP1 subjected to the blast loading produced by the 7.68 g TNT charge. A mirror of the current numerical model is also shown.

**Figure 12:**
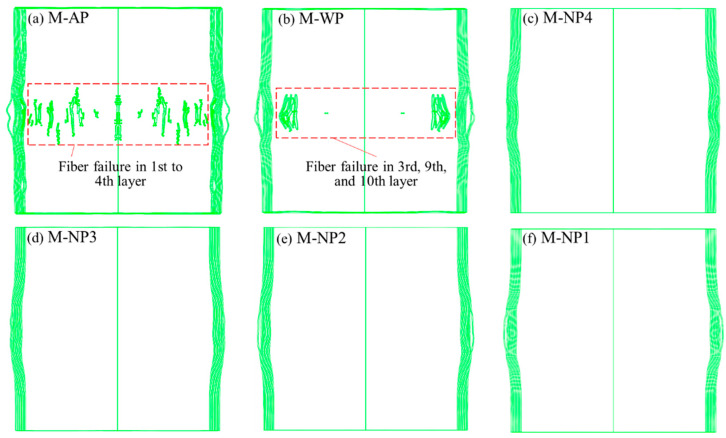
Overall distributions of fiber failure in the fiber circles of structures: (**a**) AP, (**b**) WP, (**c**) NP4, (**d**) NP3, (**e**) NP2, and (**f**) NP1 0.1 ms after detonation of 7.68 g of TNT, as seen in the A-A section defined in Figure 11. The model results are displayed in transparent mode such that all the fiber failure cracks are visible in the images.

Subjected to the blast loading from a 7.68 g TNT charge, the fiber circles of these structures experienced distinct deformation and failure characteristics. The dynamic responses of structures AP, WP, and NP1 are shown in Figure 11, and the overall distributions of fiber failure are shown in Figure 12. Without protection by any liquid, the fibers failed in the first four fiber layers of structure AP within 0.25 ms after detonation, which formed several evident cracks extending along the height direction of the fiber circle (i.e., vertical cracks). Along with fiber failure, localized radial bulges developed on the rear surface of the circle.

For structure WP, adding the water layer reduced both the number of failed fibers and the bulge size compared to structure AP (Figure 11b and Figure 12b). Shorter vertical cracks formed in the third, ninth, and tenth fiber layer, indicating that the water also shifted the location of high hoop stress from the layers near the blast-facing surface to the rear surface. All four NMLSs caused no fiber failure and further reduced the bulge size (Figure 11c and Figure 12c–f), indicating that the blast mitigation performance of the liquid was improved by the energy absorption. Modifying the energy absorption capacity by adjusting the *r_p_* of the NMLSs resulted in only minor differences in the overall deformation of the fiber circles. Further comparisons were conducted between the structures with respect to peak hoop stress and energy transfer.

**Figure 13:**
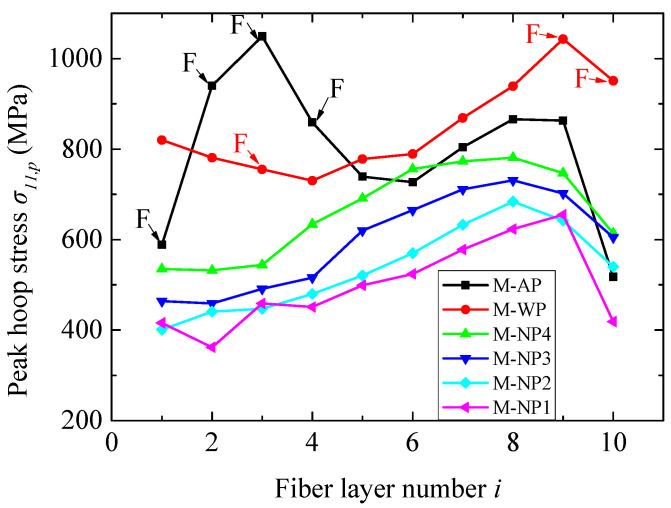
The peak hoop stresses *σ*_11,*p*_ at the gauges of each fiber layer in cases of 7.68 g of TNT. The layers containing failed fibers are marked by the letter F.

The peak hoop stresses *σ*_11,*p*_ at the gauges of each fiber layer of different structures are shown in Figure 13. Since an in-plane unloading wave would transmit into the fiber layer once some fibers failed (i.e., the corresponding elements were removed from the simulations), the increase in hoop stress in the surrounding fiber elements would be limited to some extent, depending on the distance from the removed elements. Consequently, *σ*_11,*p*_ in the layer containing failed fibers tended to underestimate the overall stress level of this layer. Hence, the reduction *R_s_* of *σ*_11,*p*_ was not employed to quantitatively estimate the blast mitigation performance, considering that fibers failed in the first four layers for the reference structure, AP. Instead, comparisons were directly made in terms of *σ*_11,*p*_. As shown in Figure 13, the water layer reduced *σ*_11,*p*_ in the layers near the blast-facing surface but increased it in the layers near the rear surface compared to structure AP, consistent with the difference between the WP and AP in the fiber failure location. These results demonstrate that the benefits of simply increasing the structural weight for blast mitigation are limited. In contrast, all four NMLSs reduced *σ*_11,*p*_ in almost all the layers, exhibiting much better blast mitigation performance than the water. Furthermore, the magnitude of *σ*_11,*p*_ basically increased with the increase of *r_p_*, i.e., a NMLS with greater energy absorption capacity had better blast mitigation. The influence of *r_p_* on the magnitude of *σ*_11,*p*_ is completely reversed compared to the case using a 3.80 g TNT charge.

**Figure 14:**
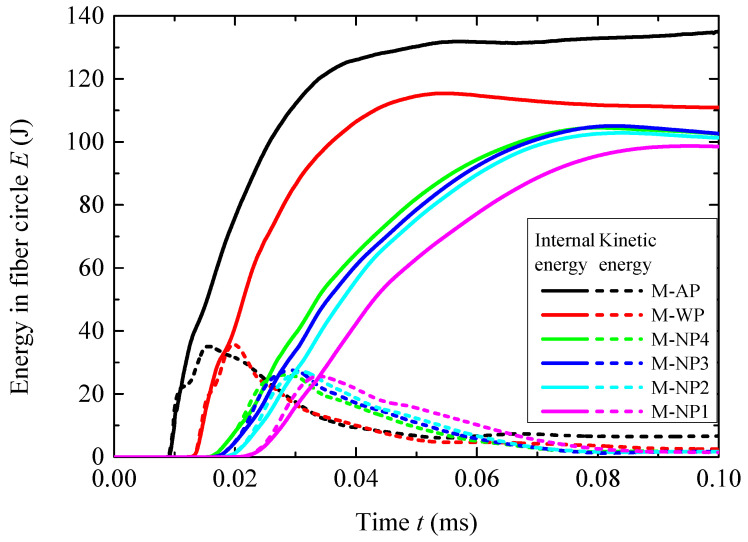
Histories of the internal energy *E_i_* and kinetic energy *E_k_* in the fiber circles for cases of 7.68 g of TNT.

The overall blast mitigation performance was evaluated based on energy transfer. The histories of the total internal energy *E_i_* and kinetic energy *E_k_* obtained by the fiber circle are shown in Figure 14. The peaks *E_ip_* and *E_kp_*, along with their reductions *R_i_* and *R_k_* relative to structure AP, are compared in Table 6. Structure WP had an *R_i_* of 12.5% and an *R_k_* of −1.7%, indicating that the water layer reduced the total energy transferred into the fiber circle and remarkably altered the proportion of internal energy and kinetic energy. All the NMLSs outperformed the water in overall blast mitigation performance; the values of *R_i_* and *R_k_* for these NMLSs ranged from 20.3% to 25.2% and from 21.7% to 26.8%, respectively. Both *R_i_* and *R_k_* first decreased as *r_p_* increased from 1:4 to 1:3 and then increased as it further increased to 1:1. The overall trend is consistent with the variations in the reduction in hoop tensile stress; i.e., increasing the energy absorption capacity of the NMLS improved the blast mitigation performance.

**Figure 15:**
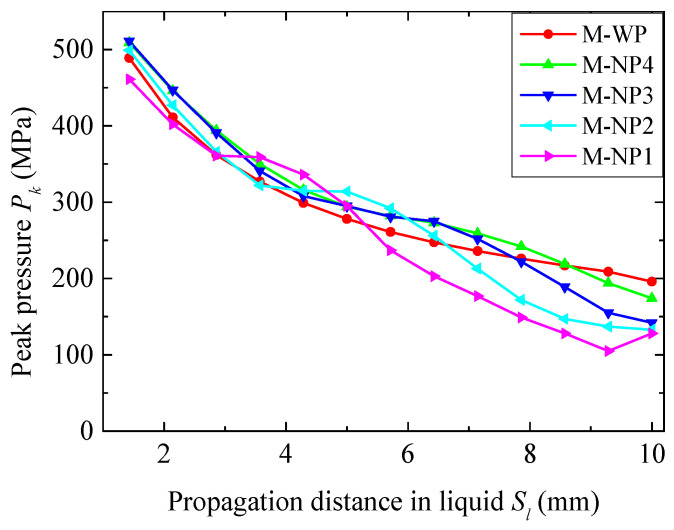
Attenuation of the peak pressure *P_k_* in the different liquids in the cases using a 7.68 g TNT.

The peak blast pressures *P_k_*, varying with propagation *S_l_* in the different liquids, are compared in Figure 15. Due to a significant backward flow of the liquid on the blast-facing surface as the pressure wave passed through, the *P_k_* reading at the first gauge is omitted. At the first several millimeters of the water, *P_k_* decayed at a rate comparable to that in the NMLSs. As *P_k_* entered the slowing phase of the NMLSs, the decay rate in the water became greater than that in the NMLSs. This trend reversed once *P_k_* exited the slowing phase. As a result, all four NMLSs had smaller *P_k_* than the water when approaching the liquid–fiber interface; e.g., at *S_l_* = 10 mm, *P_k_* for N1 (128 MPa) was 34.7% lower than that for water (196 MPa). The overall decay rate of the NMLSs increased in the order of N4, N3, N2, and N1, consistent with the results in the cases using a 3.68 g TNT. Increasing *r_p_* increased the energy absorption capacity and thus caused faster pressure attenuation.

It should be noted that *P_k_* near the liquid–fiber interface significantly exceeded the full-compaction pressure (27.5 MPa) of the NMLS. Hence, the NMLSs were in the densification state and had the same wave speed of 1500 m/s. However, the densities of the NMLSs after densification increased in the order of N4, N3, N2, and N1. As a result, the wave impedance after densification increased as the *r_p_* increased. Therefore, in the current cases, using a 7.68 g TNT, a NMLS with greater energy absorption capacity exhibited faster pressure attenuation and larger wave impedance, which collectively contributed to the superior blast mitigation performance.

### 3.3. Using the TNT Charge of 11.52 g

**Figure 16:**
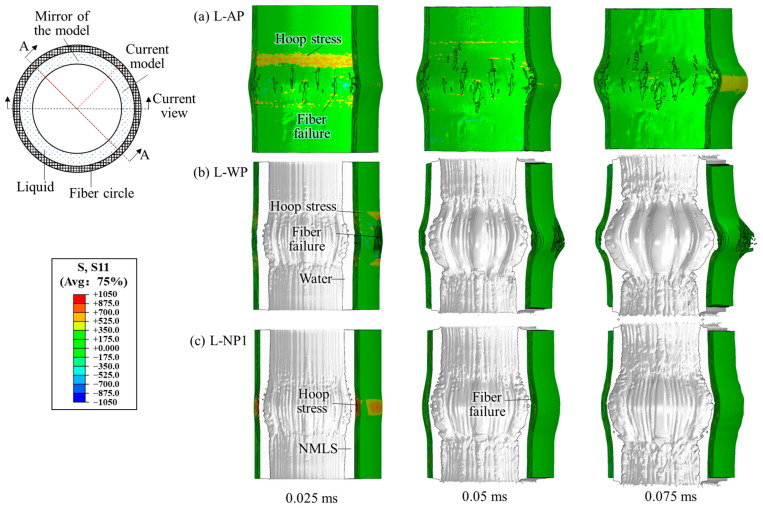
Dynamic response of the structures (**a**) AP, (**b**) WP, and (**c**) NP1 subjected to the blast loading produced by the 11.52 g TNT charge. A mirror of the current numerical model is also shown.

The blast loading became much more intense as the charge mass increased to 11.52 g. All six different structures experienced remarkable fiber failure. The dynamic responses of structures AP, WP, and NP1 are shown in Figure 16. For structure AP, the fibers failed successively before 0.05 ms, and only the outermost fiber layer remained intact. For structure WP, adding a water layer did not help reduce the number of failed layers. On the contrary, no fiber layer remained intact. By replacing the water with N1, the onset of fiber failure was delayed until after 0.025 ms, and the outermost fiber layer remained intact. In contrast, for NP4, NP3, and NP2, all fiber layers experienced failure, similar to that observed in structure WP.

**Figure 17:**
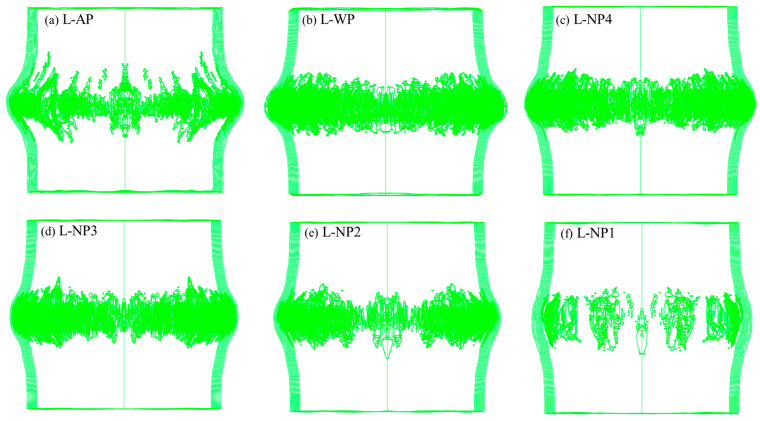
Overall distributions of fiber failure in the fiber circles of structures: (**a**) AP, (**b**) WP, (**c**) NP4, (**d**) NP3, (**e**) NP2, and (**f**) NP1 0.075 ms after detonation of 11.52 g of TNT, as viewed in the A-A section defined in Figure 16. The model results are displayed in transparent mode such that all the fiber failure cracks are visible in the images.

The overall distributions of fiber failure in the fiber circles of different structures are shown in Figure 17. Compared to AP, the crack widths in the fiber circles of the other structures containing liquids covered a narrower height range. This difference is likely due to the liquid causing the blast loading on the fiber circle to become more concentrated. The crack distributions in structures WP, NP4, NP3, and NP2 showed limited variations, with their cracks appearing denser than those in AP. In NP1, however, slightly fewer cracks developed, which can be attributed to N1’s greater energy absorption capacity.

Except in the outermost layers of AP and NP1, the peak hoop stress in the fiber circles reached or was slightly below the dynamic fracture strength (1050 MPa). Based on these hoop stress results, the liquids provided minimal blast mitigation in the current cases using an 11.52 g charge. Regarding the energy transferred into the fiber circles, the internal and kinetic energy continued to increase even after 0.075 ms, by which time significant fiber failure had already occurred. The further increase in energy corresponded to the escalation of global deformation, such as bulging and out-of-plane shearing. Since fiber failure is the primary indicator of blast mitigation effectiveness, a further comparison of energy peaks is not made.

**Figure 18:**
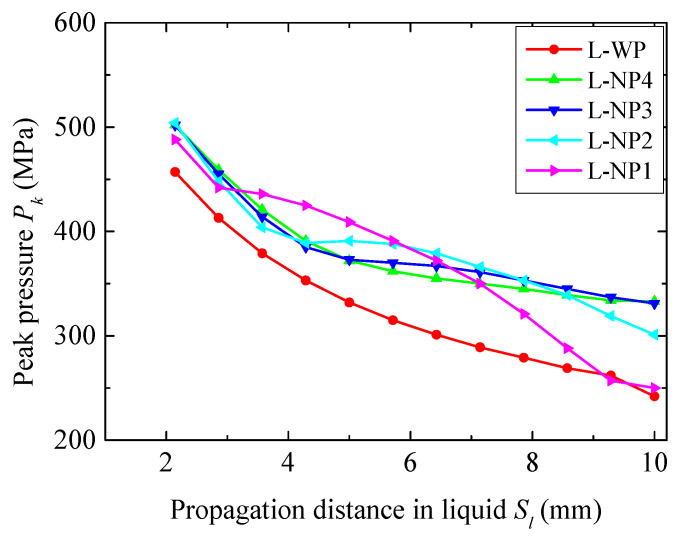
Attenuation of the peak pressure *P_k_* in the different liquids in the cases using a 11.52 g TNT.

The peak blast pressures *P_k_*, varying with propagation *S_l_* in the different liquids, are compared in Figure 18. Increasing the charge mass from 7.68 g to 11.52 g further intensified the blast loading, causing the liquids to flow backward and exit the first two layers of elements before the pressure reached its peak in these elements. As a result, the *P_k_* readings at the first two gauges were omitted. The overall decay rate of *P_k_* in water was greater than that in N4, N3, and N2, which contrasts with the results observed when using a smaller charge mass. This reversed outcome highlights another factor affecting pressure attenuation in the liquids: the influence of unloading waves. Due to the inelastic nature of the infiltration process, the transmission of the unloading waves from the blast-facing surface of the NMLS was delayed, which hindered pressure attenuation. This delay is likely the primary cause of the slower pressure decay in N4, N3, and N2, despite their energy absorption capabilities, compared to water. In N1, *P_k_* decayed more slowly than in water before *S_l_* = 5 mm, but significantly faster thereafter due to N1’s exceptional energy absorption capacity. Near the liquid–fiber interface, water exhibited the lowest *P_k_* of 242 MPa. *P_k_* in the NMLSs decreased as the *r_p_* increased, consistent with the results in the cases with smaller mass charges. The *P_k_* in N1 (250 MPa) was slightly higher than that in water. Meanwhile, after densification, N1 exhibited a greater wave impedance, due to its higher density, compared to water. This explains why fewer fibers failed in the fiber circle of NP1 compared to WP.

## 4. Conclusions

This investigation numerically evaluated the effectiveness of NMLSs in reducing the blast effects on a fiber–composite circle. The numerical models of a liquid–fiber structure subjected to internal, close-field blast loading from a TNT explosive charge were established using the CEL method. Three different types of structures, fiber circle alone, water–fiber circle, and NMLS–fiber circle, were considered. Comparisons were made between the dynamic responses of the fiber circles in these structures to quantitatively evaluate the benefits derived from the energy absorption capacity of the NMLS.

A commonly studied NMLS, Fluka 100-C8 nanoporous silica particles suspended in distilled water, was chosen as the research objective. Four different mass ratios of particles to water, 1:4, 1:3, 1:2, and 1:1, were considered in the simulations to vary the energy absorption capacity of the NMLSs. These NMLSs maintained a density of 1 g/cm^3^ regardless of the ratio, which ensured that all the NMLSs and water had the same mass in the different liquid–fiber structures to make a fair comparison. The NMLSs were modeled using EOS compaction, which was validated through quasi-static compression tests, to phenomenologically describe the infiltration of water into nanopores.

Three different masses, 3.80, 7.68, and 11.52 g, of TNT charges were used to evaluate the blast mitigation performance of those liquids under different levels of blast loadings. Subjected to blast loading from the 3.80 g charge, all the fiber circles in the different structures remained intact. Both the water and NMLSs significantly mitigated the blast effects on the fiber circles. Adding the water layer reduced the peak hoop stress in the fiber layers by 0.24–39.1%. Replacing water with NMLSs increased these reductions to 19.9–45.0%. The superior performance of NMLSs over water can be attributed to their energy absorption capacity, which causes better attenuation of blast pressure. Specifically, the peak pressure in the NMLSs was only 18.4% of that in the water when approaching the liquid–fiber interface. All four NMLSs could rapidly reduce the blast pressure to the infiltration pressure (17 MPa) of the NMLSs, but their wave impedances decreased as the particle-to-water ratio increased, resulting in a NMLS with a greater energy absorption capacity, however, with inferior blast mitigation performance.

When the charge mass was increased to 7.68 g, the first four layers of the fiber circle experienced significant fiber failure without any liquid protection. Adding a water layer significantly reduced the peak hoop stress in these front layers but increased it in the rear layers, still resulting in the failure of three layers. In contrast, replacing the water with NMLSs substantially reduced the hoop stress throughout the entire fiber circle, and all the fiber layers remained intact. This improvement can be attributed to the greater attenuation of blast pressure due to the energy absorption capacity of the NMLSs. The blast mitigation performance improved as the particle-to-water ratio increased, which was in stark contrast to the results observed with a 3.80 g TNT charge. This reversed trend can be attributed to the transition of NMLSs’ response from the infiltration stage to the densification stage near the liquid–fiber interface as blast loading increased. During the densification phase, NMLSs with a higher particle-to-water ratio resulted in a lower blast pressure and exhibited greater wave impedance, which collectively contributed to the superior blast mitigation performance.

When the charge mass was further increased to 11.52 g, all the fiber circles experienced significant fiber failure. Only the NMLS with the highest energy absorption capacity showed slight blast mitigation, as evidenced by fewer failure cracks. The pressure attenuation rate in the NMLS was generally lower than that in water, indicating that the energy absorption capacity of the NMLS was insufficient to handle such intense blast loading and made only minimal contributions to blast mitigation.

These results demonstrate that the blast mitigation performance of a NMLS depends not only on its energy absorption capacity but is also influenced by wave impedance, which varies significantly with the NMLS’s state. Efficiently utilizing a NMLS for blast mitigation requires carefully matching the protective structure to the blast-loading conditions.

## Figures and Tables

**Table 1:** Schemes of the numerical simulations.

No.	Name	Charge Size (mm)	Charge Mass (g)	Liquid	Liquid Mass (g)	Fiber Mass (g)
1	S-AP	Φ25 × 5	3.80	None	0	146.4 g
2	S-WP			Water	249.4	
3	S-NP1			N1		
4	S-NP2			N2		
5	S-NP3			N3		
6	S-NP4			N4		
7	S-AP	Φ25 × 10	7.68	None	0	
8	M-WP			Water	249.4	
9	M-NP1			N1		
10	M-NP2			N2		
11	M-NP3			N3		
12	M-NP4			N4		
13	L-AP	Φ25 × 15	11.52	None	0	
14	L-WP			Water	249.4	
15	L-NP1			N1		
16	L-NP2			N2		
17	L-NP3			N3		
18	L-NP4			N4		

**Table 2:** Material parameters of the NMLS and water for the numerical models.

Particle–Water Ratio *r_p_*	EOS *Us*–*Up*		EOS Compaction			
	Reference Density *ρ*_0_ (g/cm^3^)	Reference Sound Speed *c*_0_ (m/s)	Reference Sound Speed in the Porous Material *c_p_*_0_ (m/s)	Porosity *n*_0_	Infiltration Pressure *P*_in_ (MPa)	Compaction Pressure at Which All Nanopores Are Filled with Water *P*_c_ (MPa)
1:1	1.377	1500	400	0.274	17	27.5
1:2	1.224	1500	500	0.183	17	27.5
1:3	1.159	1500	800	0.137	17	27.5
1:4	1.124	1500	1000	0.110	17	27.5
0 (Water)	1	1500	-	-	-	-

**Table 3:** Material properties for air [35].

Parameter	Density *ρ*	Gas Constant *R*	Air Pressure *P*_0_	Specific Heat *C*_v_
Value	1.225 kg/m^3^	287.058 J/(kg·K)	0.101325 MPa	718 J/(kg·K)

**Table 4:** Material properties for TNT [34].

Parameter	Density *ρ*	Detonation Velocity *D*	Fitting Coefficient					Detonation Energy Density *e*
			A	B	ω	R_1_	R_2_	
Value	1630 kg/m^3^	6930 m/s	373.77 GPa	3.75 GPa	0.35	4.15	0.9	3,681,000 kJ/kg

**Table 5:** Peak internal and kinetic energies in the fiber circles for cases of 3.80 g of TNT.

Name	Peak Internal Energy *E_ip_* (J)	Peak Kinetic Energy *E_kp_* (J)	Reduction *R_i_* in *E_ip_* (%)	Reduction *R_k_* in *E_kp_* (%)
S-AP	17.8	4.36	-	
S-WP	11.8	3.35	33.7	23.2
S-NP4	7.97	2.54	55.2	41.7
S-NP3	8.61	2.77	51.6	36.5
S-NP2	9.84	3.12	44.7	28.4
S-NP1	10.4	3.25	41.6	25.5
S-NP1a	7.56	2.24	57.5	48.6

**Table 6:** Peak internal and kinetic energies in the fiber circles for cases of 7.68 g of TNT.

Name	Peak Internal Energy *E_ip_* (J)	Peak Kinetic Energy *E_kp_* (J)	Reduction *R_i_* in *E_ip_* (%)	Reduction *R_k_* in *E_kp_* (%)
M-AP	131.9	35.1	-	-
M-WP	115.4	35.7	12.5	−1.7
M-NP4	104.5	26.1	20.8	25.6
M-NP3	105.1	27.5	20.3	21.7
M-NP2	102.8	27.1	22.1	22.8
M-NP1	98.7	25.7	25.2	26.8

## Data Availability

The raw/processed data required to reproduce these findings cannot be shared at this time as the data also form part of an ongoing study.

## Abbreviations

Abbreviation	Full Form/meaning
AP	Fiber-ply alone structure
CEL	Coupled Eulerian–Lagrangian
EASs	Energy absorption systems
EOS	Equation of state
IEDs	Improvised explosive devices
JWL	Jones–Wilkins–Lee
NMLS	Nanoporous material liquid system
NMLSs	Nanoporous material liquid systems
N1	NMLS with a particle–water mass ratio of 1:1
N2	NMLS with a particle–water mass ratio of 1:2
N3	NMLS with a particle–water mass ratio of 1:3
N4	NMLS with a particle–water mass ratio of 1:4
NP	NMLS fiber-ply structure
NP1	NP with NMLS N1
NP2	NP with NMLS N2
NP3	NP with NMLS N3
NP4	NP with NMLS N4
UHMWPE	Ultra-high-molecular-weight polyethylene
WP	Water fiber-ply structure
